# Advances in the Research of Hypoxia-Inducible Factor-1Alpha and Plasminogen Activator Inhibitor-1 in Vascular-Related Diseases

**DOI:** 10.14740/jocmr6330

**Published:** 2026-03-26

**Authors:** Xiao Xu, Xiao Hu Ge

**Affiliations:** a Xinjiang Medical University Graduate School, Urumqi, Xinjiang Uygur Autonomous Region, Urumqi 831500, China; b Department of Vascular Surgery, People’s Hospital of Xinjiang Uygur Autonomous Region, Urumqi, Xinjiang Uygur Autonomous Region, Urumqi 831500, China

**Keywords:** HIF-1α, PAI-1, Vascular remodeling, Hypoxia signal, Fibrinolysis system, Targeted therapy

## Abstract

Hypoxia-inducible factor-1α (HIF-1α) and plasminogen activator inhibitor-1 (PAI-1) play crucial roles in vascular homeostasis and pathological remodeling. Their intertwined regulatory network represents a focal point in vascular disease research. Under physiological conditions, HIF-1α and PAI-1 act coordinately to maintain vascular adaptability and repair capacity. However, under pathological conditions such as hypoxia or metabolic dysregulation, their aberrant activation constitutes a significant driver of vascular pathologies, including thrombosis, fibrosis, and atherosclerosis. Consequently, targeting this regulatory network may offer novel therapeutic approaches for vascular diseases. Cellular exposure to stressors such as hypoxia or inflammation induces the expression of key regulatory factors including HIF-1α and PAI-1. As the principal transcription factor mediating hypoxic responses, HIF-1α activates downstream target genes such as vascular endothelial growth factor (VEGF) and glucose transporter 1 (GLUT1), thereby regulating angiogenesis, metabolic reprogramming, and inflammatory responses. PAI-1, a serine protease inhibitor, contributes critically to thrombosis, fibrosis, and endothelial dysfunction through inhibition of the fibrinolytic system and modulation of extracellular matrix (ECM) degradation.

## Introduction

Vascular diseases exhibit high global prevalence with continuously rising incidence, placing substantial economic burdens on societies and families. Consequently, a comprehensive investigation into the relationship between vascular diseases and hypoxia-inducible factor-1α (HIF-1α) and plasminogen activator inhibitor-1 (PAI-1) is crucial for advancing research in this field and establishing a theoretical foundation for understanding their pathogenesis.

The research landscape of the HIF-1α/PAI-1 axis is built upon pivotal historical discoveries. The field was inaugurated by the seminal identification of HIF-1 in the early 1990s, which established the molecular basis for oxygen sensing [1]. Subsequent research throughout the following decades elucidated HIF-1α’s central role in vascular biology, including angiogenesis and metabolic adaptation [2]. In parallel, the critical pathological function of PAI-1 as a key regulator of fibrinolysis and fibrosis in cardiovascular contexts was firmly established [3]. It is the more recent convergence of these two research trajectories that has spotlighted their intricate interplay within the hypoxic microenvironment as a major focus for understanding vascular disease mechanisms.

HIF-1α, a core transcriptional regulator in the cellular response to hypoxic microenvironments, exhibits biologically significant interactions with PAI-1 in the regulation of vascular homeostasis. In recent years, the molecular mechanisms underlying the interplay between HIF-1α and PAI-1, along with their synergistic roles in regulating the pathophysiology of various diseases, have become a major research focus. This article systematically reviews the structural and functional characteristics of the HIF-1α/PAI-1 axis and its molecular associations with vascular diseases, aiming to provide a solid theoretical basis for elucidating their pathological mechanisms.

### Concept and function of HIF-1α

The molecular characteristics and functional mechanisms of HIF-1 were first elucidated by Semenza’s research team in 1992. HIF-1 functions as a heterodimer, comprising an oxygen-sensitive α subunit (HIF-1α, ∼120 kDa) and a constitutively expressed β subunit (HIF-1β, ∼91-94 kDa), with its biological activity primarily regulated by the α subunit [4]. Both subunits contain a conserved basic helix-loop-helix (bHLH) domain essential for DNA binding [5]. Research confirms that HIF-1α directly regulates a cluster of target genes—including erythropoietin (EPO), vascular endothelial growth factor (VEGF), and glucose transporter 1 (GLUT1)—all harboring conserved HIF-1α binding motifs in their regulatory regions. This observation establishes HIF-1α as a pivotal regulator within the hypoxia-adaptive transcriptional network. By orchestrating the reprogramming of multiple gene expression profiles, HIF-1α mediates critical physiological and pathological processes such as cellular metabolism, angiogenesis, and oxygen homeostasis, underscoring its central role in hypoxic signaling [6]. Furthermore, HIF-1α integrates oxygen-sensing pathways with epigenetic regulatory mechanisms to coordinate transcriptional reprogramming of glycolytic metabolism, angiogenesis, and erythropoiesis, thereby playing a core role in ischemic injury repair, tumor microenvironment adaptation, and oxygen homeostasis maintenance. Critically, the oxygen-dependent degradation domain (ODD) of HIF-1α and its interaction with prolyl hydroxylase (PHD) solidify its function as a molecular switch for dynamic oxygen signaling regulation.

### Concept and function of PAI-1

PAI-1 is a serine protease inhibitor encoded by the *SERPINE1* gene. It acts as the primary inhibitor of both urokinase-type plasminogen activator (uPA) and tissue-type plasminogen activator (tPA), thereby inhibiting fibrinolysis. PAI-1 is a single-chain glycoprotein with a molecular weight of approximately 45 kDa, composed of 379–381 amino acid residues. It contains nine α-helices and three β-sheet structures. The functional domains of PAI-1 include the reactive center loop (RCL), which mediates binding to plasminogen activators; a flexible hinge region involving helices D (hD), E (hE), and F (hF) that binds to vitronectin; and regions hD and hE, which also participate in the binding of low-density lipoprotein receptor-related protein (LRP) [7]. Depending on its source and characteristics, PAI can be classified into several types, including endothelial PAI (PAI-1), placental PAI (PAI-2), urinary PAI (PAI-3), and protease-nexin-1 (PAI-4), among which PAI-1 plays the most critical role in physiological processes [8]. PAI-1 is primarily secreted into the plasma in its active form, and its physiological functions are mainly manifested in the following aspects: by inhibiting the activities of uPA and tPA, it affects various cellular functions such as nerve growth, collagen activation, and tissue repair; during inflammatory responses, complement activation, and coagulation processes, PAI-1 exerts important inhibitory and degradative effects on relevant proteins; it protects cell membrane helices from degradation by plasma enzymes; it maintains cell-to-cell contact surfaces, thereby ensuring tissue integrity; and within the cell cycle, the intracellular accumulation of active PAI-1 and changes in its transcription are crucial for maintaining cell morphology, cell proliferation, cell adhesion to the matrix, signal transduction, and gene expression [9].

### Relationship between HIF-1α and PAI-1

**Figure 1:**
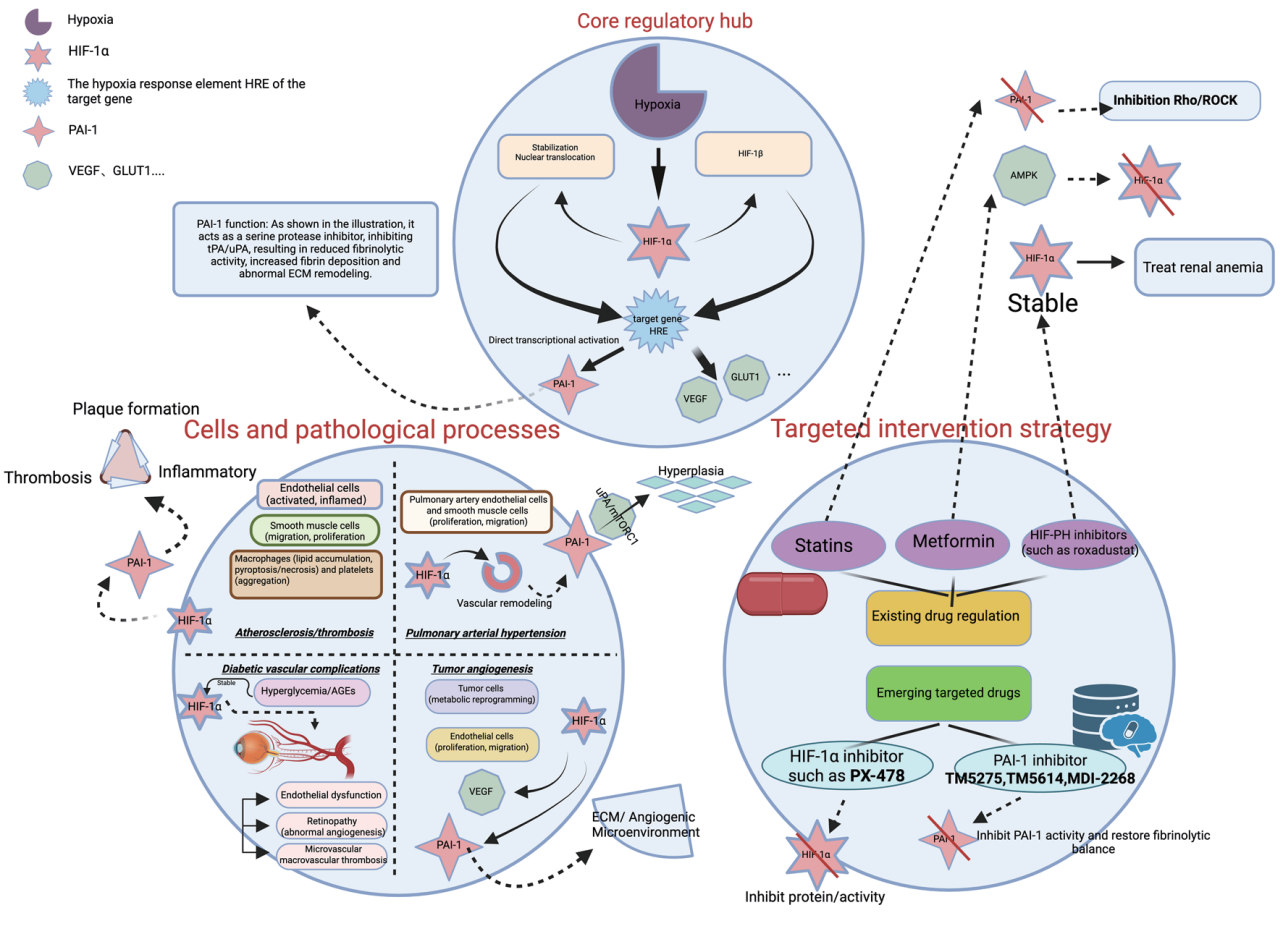
The HIF-1α/PAI-1 regulatory network in vascular diseases. Under hypoxic conditions, HIF-1α is stabilized and translocates to the nucleus, where it transcriptionally activates target genes including PAI-1, VEGF, and GLUT1 by binding to hypoxia response elements (HREs). Elevated PAI-1 contributes to the pathogenesis of various vascular disorders—such as atherosclerosis/thrombosis, pulmonary arterial hypertension (PAH), diabetic vascular complications, and tumor angiogenesis—through mechanisms involving fibrinolysis inhibition, inflammatory modulation, extracellular matrix (ECM) remodeling, and vascular remodeling. The diagram also summarizes current targeted intervention strategies, including existing drugs (e.g., statins) and emerging inhibitors targeting HIF-1α (e.g., PX-478) or PAI-1 (e.g., TM5275, TM5614, MDI-2268). HIF-1α: hypoxia-inducible factor-1α; PAI-1: plasminogen activator inhibitor-1. VEGF: vascular endothelial growth factor; GLUT1: glucose transporter 1.

HIF-1α and PAI-1 engage in complex interregulatory mechanisms across various pathophysiological processes. Under hypoxic conditions, HIF-1α binds to the hypoxia response element (HRE) within the PAI-1 gene promoter, thereby activating PAI-1 transcription [10]. For instance, hypoxia stabilizes HIF-1α within the supernatant of alveolar macrophages and enhances its induction of PAI-1 [11]. Conversely, PAI-1 provides indirect feedback regulation of HIF-1α activity by modulating extracellular matrix (ECM) remodeling and cellular signaling pathways. Specifically, PAI-1 inhibits uPA and tPA, reducing plasmin generation. This, in turn, diminishes ECM degradation and promotes fibrin deposition, fostering a hypoxic microenvironment that activates HIF-1α [12]. The synergistic effects of HIF-1α and PAI-1 are particularly evident in pathological contexts. During tumor progression, hypoxia upregulates PAI-1 via HIF-1α, promoting tumor angiogenesis [13]. In thrombosis, HIF-1α-mediated hypoxic upregulation of PAI-1 may contribute to atherosclerotic plaque stability and increases the risk of thrombus formation [14]. Experimental studies demonstrate that hypoxia enhances HIF-1α stability and activity, significantly inducing PAI-1 expression in human hepatocellular carcinoma cells (HepG2 cells) and elucidating the mechanism underlying hypoxia-induced PAI-1 upregulation. Within this mechanism, HIF-1α and PAI-1 act synergistically: PAI-1 inhibits the fibrinolytic process, reducing fibrinolysis and promoting thrombus formation [15]. The synergistic regulatory interplay between HIF-1α and PAI-1 is summarized in Figure 1.

## The Role of HIF-1α and PAI-1 in Vascular Diseases

### The multifaceted roles of HIF-1α and PAI-1 in atherosclerosis

The pathogenesis of atherosclerosis involves a complex interplay of molecular signals, with HIF-1α and PAI-1 serving as pivotal, interconnected regulators. The process is initiated at sites predisposed to plaque formation, such as arterial branches characterized by low shear stress. Under these conditions, endothelial HIF-1α expression is upregulated via transcriptional activation by nuclear factor-κB (NF-κB) and protein stabilization mediated by the deubiquitinase Cezanne (also known as OTUD7B). This early HIF-1α activation drives endothelial metabolic reprogramming and inflammatory responses, establishing a pro-atherogenic milieu [16].

Subsequent hypoxia within the evolving plaque further amplifies HIF-1α signaling, which orchestrates key cellular events. In vascular smooth muscle cells (VSMCs), HIF-1α directly binds to the promoter of the Kruppel-like factor 4 (*KLF4*) gene, inducing its expression. This HIF-1α-mediated KLF4 upregulation activates pro-migratory signaling pathways, enhancing VSMC migration—a critical step in vascular remodeling and neointima formation [17]. Concurrently, within macrophages, HIF-1α activation shifts cellular metabolism by reducing oxidative phosphorylation and ATP levels while elevating reactive oxygen species (ROS), thereby promoting necroptosis. This process is fine-tuned by HIF-1α through the upregulation of miR-210 and downregulation of miR-383, with the latter targeting poly(ADP-ribose) glycohydrolase (PARG) to influence DNA damage repair pathways, collectively affecting plaque cellularity and stability [18]. The therapeutic relevance of targeting macrophage HIF-1α is underscored by the action of salidroside (a bioactive compound derived from Rhodiola rosea), a natural compound that ameliorates macrophage lipid accumulation and reduces atherosclerotic plaques by inhibiting HIF-1α-induced pyroptosis [19].

In parallel, PAI-1 expression is markedly elevated in atherosclerotic lesions and is functionally linked to inflammatory pathways (e.g., NF-κB signaling) and oxidative stress [20]. PAI-1 contributes to disease progression through multiple mechanisms: it promotes ox-LDL-induced senescence in aortic endothelial cells and macrophages via p16/p21 and p53-mediated cell cycle arrest [21]; its genetic deficiency or pharmacological inhibition in animal models significantly reduces lesion size by dampening macrophage infiltration, inhibiting VSMC proliferation/migration, and mitigating oxidative stress [22]. The anti-atherosclerotic effects of agents like baicalein are partly attributable to the inhibition of PAI-1 activity, leading to attenuated endothelial damage and suppressed thrombosis [20].

A critical mechanistic link in atherosclerosis is the HIF-1α-mediated upregulation of PAI-1. Within the hypoxic plaque microenvironment, HIF-1α activation directly increases the expression of pro-thrombotic factors, including PAI-1 [14]. This regulatory relationship, modulated by hemodynamic forces, results in suppressed fibrinolysis and accelerated thrombotic complications [23]. The clinical correlation of this pathway is evidenced by the increased presence of HIF-1α-immunopositive cells within coronary artery plaques, particularly in areas of thrombosis [14]. The interconnected nature of the HIF-1α and PAI-1 pathways is further highlighted by the action of silymarin, which attenuates atherogenesis by inhibiting HIF-1α-mediated pyroptosis [19]. In summary, HIF-1α and PAI-1 form a synergistic regulatory network that coordinates endothelial dysfunction, smooth muscle cell migration, macrophage inflammation and death, and ultimately thrombosis, driving the progression of atherosclerosis.

### Pulmonary arterial hypertension (PAH)

#### HIF-1α drives pulmonary vascular remodeling and hypoxic adaptation

Research has established that calpain-1 exacerbates pulmonary vascular remodeling and fibrosis, thereby promoting the development of PAH. This occurs through NF-κB (p65)-mediated upregulation of HIF-1α expression under hypoxic conditions [24]. Hypoxia induces increased expression of connexin 43 (Cx43) and enhanced phosphorylation at its Ser368 site in both rat pulmonary arteries and pulmonary arterial smooth muscle cells (PASMCs). These changes promote PASMC proliferation and migration. The HIF-1α inhibitor actinomycin D attenuates this proliferative/migratory effect. Furthermore, chromatin immunoprecipitation assays confirmed direct binding of HIF-1α to the Cx43 promoter, demonstrating that HIF-1α acts as an upstream regulator enhancing Cx43 expression. Collectively, these findings indicate that the HIF-1α/Cx43 axis plays a critical role in hypoxia-induced PAH pathogenesis. Modulation of this axis may therefore represent a therapeutic strategy for controlling pulmonary vascular remodeling [25].

#### The unique role of PAI-1 deficiency in PAH pathogenesis

PAH patients exhibit deficient PAI-1 expression in the smooth muscle compartment of remodeled small pulmonary arteries (PAs) and in early-passage PASMCs compared to non-PAH controls. Both male and female PAI-1 knockout (PAI-1^−/−^) mice develop spontaneous pulmonary vascular remodeling and PAH, characterized by increased pulmonary arterial medial thickness, elevated systolic right ventricular pressure, and right ventricular hypertrophy [26]. Mechanistically, PAI-1 deficiency in PAH subjects potentiates excessive PASMC proliferation and vascular remodeling through unopposed uPA activity, driving mTORC1 and TGF-β signaling upregulation [27]. This deficiency is clinically relevant: PAH patients demonstrate significantly reduced serum and lung tissue PAI-1 levels alongside elevated uPA, correlating with disease progression [28].

#### Therapeutic outlook: targeting the hypoxia-fibrinolysis crosstalk

The contrasting roles of HIF-1α (promoter) and PAI-1 (potential suppressor) in PAH highlight the complexity of vascular remodeling. Future therapeutic strategies may need to consider the balance between hypoxic signaling and fibrinolytic activity. Simultaneous modulation of the HIF-1α pathway and restoration of local PAI-1 activity or its downstream effects could represent a novel approach to stabilizing the pulmonary vasculature.

### Thrombosis

#### PAI-1: the core regulator of fibrinolytic balance and thrombotic risk

In acute cerebral infarction, serum anti-PAI-1 antibody levels are significantly elevated and independently associated with established vascular risk factors (age, sex, hypertension, diabetes, cardiovascular disease) and carotid intima-media thickness. Multivariate analysis confirms anti-PAI-1 antibodies as independent predictors of acute ischemic stroke [29]. Complementing this, a population-based nested case-control study established a dose-response relationship between plasma PAI-1 levels and future venous thromboembolism (VTE) risk. Notably, PAI-1 mediates approximately 15% of VTE risk in obese individuals, representing the first epidemiological evidence linking elevated PAI-1 to incident VTE [30]. Therapeutically, Danshen-Honghua injection ameliorates thrombotic risk by reducing PAI-1 levels and restoring fibrinolytic homeostasis, demonstrating both PAI-1’s mechanistic role in thrombosis and its potential as a therapeutic target for anticoagulation enhancement [31].

#### HIF-1α: shaping the pro-thrombotic pathological microenvironment

This regulatory axis extends to dermal thrombosis, where mechanical compression induces hypoxia-mediated HIF-1α activation and PAI-1 induction, potentiating local fibrin deposition. HIF-1 inhibitors abolish this prothrombotic phenotype while reducing PAI-1 transcription [32].

#### The hypoxia-HIF-1α-PAI-1 pathway: a common axis in thrombosis

A critical mechanistic link in atherothrombosis is the HIF-1α-mediated upregulation of PAI-1. Within hypoxic environments, such as an evolving atherosclerotic plaque or compressed tissue, HIF-1α activation directly increases the expression of pro-thrombotic factors, including PAI-1 [14, 23]. This regulatory relationship results in suppressed fibrinolysis and accelerated thrombotic complications. In diabetic retinopathy, retinal hypoxia concurrently activates HIF-1α and PAI-1, which collectively promote thrombosis, pathological angiogenesis, and inflammation to drive disease progression. Thus, the hypoxia-HIF-1α-PAI-1 pathway serves as a fundamental mechanism connecting local tissue ischemia to systemic thrombotic risk across various vascular pathologies.

### Diabetic vascular complications

#### PAI-1 mediates microvascular and macrovascular complications

Clinical investigations demonstrate elevated PAI-1 levels in both serum and tear fluid of diabetic retinopathy (DR) patients compared to diabetic controls without DR and healthy subjects. Notably, proliferative DR (PDR) patients exhibit significantly higher serum PAI-1 concentrations than non-proliferative DR (NPDR) cases, establishing a strong positive correlation between PAI-1 elevation and DR severity [33]. Complementary research implicates PAI-1 in the pathogenesis of cardiovascular complications in type 2 diabetes mellitus (T2DM). Insulin resistance stimulates PAI-1 overproduction, creating a prothrombotic state that accelerates vascular disease onset and exacerbates clinical severity [34].

#### HIF-1α in diabetic chronic hypoxia and inflammation

In the diabetic state, factors like advanced glycation end products (AGEs) and oxidative stress can stabilize HIF-1α even under normoxic conditions. This contributes to chronic low-grade inflammation and aberrant angiogenesis in tissues like the retina [33].

#### Synergistic aggravation of vascular injury: a vicious cycle

Hyperglycemia and hypoxia synergistically upregulate both HIF-1α and PAI-1. This creates a vicious cycle: PAI-1 promotes thrombosis and ischemia, which in turn enhances HIF-1α stability; activated HIF-1α further transactivates PAI-1 and other inflammatory mediators. This interconnected network accelerates endothelial dysfunction, vascular occlusion, and end-organ damage in diabetes.

### Tumor angiogenesis

#### HIF-1α: the master transcriptional driver of tumor angiogenesis

In colorectal cancer, HIF-1α drives tumor angiogenesis by activating VEGF and other pro-angiogenic factors while promoting endothelial cell proliferation. Under hypoxia, HIF-1α further induces lactate uptake and stimulates angiogenesis-associated signaling pathways [35]. The ROS-ATM-CHK2 axis in the hypoxic tumor microenvironment enhances angiogenesis through HIF-1α stabilization. Specifically, CHK2 interacts with HIF-1α to inhibit its ubiquitination, thereby increasing HIF-1α stability and upregulating VEGF expression [36]. Conversely, β-hydroxybutyrate suppresses HIF-1α activity in colitis-associated tumor models, reducing angiogenic factor expression and inhibiting tumor-associated angiogenesis [37]. In TP53-mutated head and neck squamous cell carcinoma (HNSCC), HIF-1α activates the STAT3 pathway to induce VEGF expression, promoting tumor angiogenesis and lymphangiogenesis. Downregulation of HIF-1α and VEGF in this context inhibits these processes, reducing tumor recurrence and metastasis [38].

#### The complex role of PAI-1 in tumor angiogenesis and microenvironment remodeling

In cutaneous malignancies, elevated PAI-1 expression potentiates tumor angiogenesis through multifaceted mechanisms. These include: 1) inhibition of fibrinolytic activity generating pro-angiogenic fibrin matrices, 2) dysregulation of ECM remodeling, and 3) ligand-receptor interactions (e.g., with integrins) that collectively promote endothelial cell migration and neovascularization [39]. Contrastingly, in malignant pleural mesothelioma, PAI-1 exerts tumor-suppressive effects by restricting ECM remodeling and cellular migration, thereby inhibiting tumor progression independent of angiogenic stimuli [40]. Glioblastoma studies further reveal that cancer stemness marker ALDH1A3 drives angiogenesis via paracrine secretion of PAI-1 and IL-8 [41]. This evidence establishes PAI-1 as a context-dependent regulator of tumor vascularization.

#### Transcriptional activation of PAI-1 by HIF-1α: a key step in cooperative tumor vascularization

A critical functional interaction in tumors is the direct transactivation of PAI-1 by HIF-1α. Under hypoxic conditions, activated HIF-1α translocates to the nucleus and dimerizes with HIF-1β. This heterodimeric complex binds hypoxia response elements (HREs) in the PAI-1 promoter, directly transactivating PAI-1 transcription. Elevated PAI-1 suppresses proteolytic fibrinolytic activity, which consequently potentiates endothelial cell proliferation and migration, thereby facilitating tumor neovascularization [35]. This mechanism exemplifies a direct molecular synergy where HIF-1α amplifies the pro-angiogenic tumor microenvironment through PAI-1.

### Dysregulation of HIF-1α and PAI-1 in infectious diseases

#### Systemic dysregulation and immunothrombosis in sepsis

Sepsis induces profound systemic hypoxia and a cytokine storm, leading to concurrent overexpression of PAI-1 in endothelial and immune cells. This synergistic upregulation promotes the formation of “immunothrombi,” contributing to disseminated intravascular coagulation (DIC) and multiple organ failure [42].

#### Endothelialitis and thrombotic complications in COVID-19

SARS-CoV-2 infection stabilizes hypoxia-inducible factor HIF-1α, which directly upregulates the expression of the viral receptor ACE2 in host cells, thereby enhancing viral entry and replication. Concurrently, activated HIF-1α drives a pro-inflammatory cytokine storm, exacerbating pulmonary tissue damage. The study demonstrates that targeting HIF-1α reduces viral load and inflammatory responses, highlighting its potential as a therapeutic strategy for severe COVID-19 [43].

## Clinical Relevance of HIF-1α and PAI-1: Impact of Age, Sex, and Comorbidities

### Impact of age

Aging represents a significant independent risk factor for vascular diseases. This process is characterized by a decline in HIF-1α signaling responsiveness and a systemic downregulation of genes associated with the mobilization and function of endothelial progenitor cells (EPCs). In a murine model of acute hindlimb ischemia, aged mice exhibited a delayed and significantly attenuated peak in both HIF-1α mRNA and protein expression within ischemic muscle tissue compared to young mice. This was accompanied by a corresponding reduction in the expression of its key downstream pro-angiogenic factor, VEGF. These findings suggest a blunted response of EPCs to hypoxia-induced HIF-1α activation in aged individuals, leading to diminished neovascularization capacity in ischemic tissues [44]. Concurrently, aging-associated chronic low-grade inflammation and vascular stress drive a sustained increase in PAI-1 expression. Experimental studies indicate that pathological stress, such as hypertension, can directly induce excessive vascular PAI-1 expression. PAI-1 itself acts as a key mediator driving vascular aging and inflammation. In an L-NAME-induced hypertensive mouse model, the PAI-1 antagonist TM5441 significantly attenuated blood pressure elevation, improved vasodilation function, and effectively inhibited the expression of vascular senescence markers (e.g., SA-β-gal activity, p53, and p21 proteins). This study provides direct evidence that PAI-1 is not merely a biomarker of aging and inflammation but a critical effector promoting the senescent phenotype of endothelial cells and vascular inflammation [45]. Elevated PAI-1 levels are closely associated with an increased risk of atherothrombotic events prevalent in the elderly population [46].

### Impact of sex

Cardiovascular disease risk exhibits significant sexual dimorphism. The effects of estrogen display considerable complexity, depending on receptor subtypes and cellular context. Research has found that estrogen receptor β can significantly inhibit the transcriptional activity of HIF-1 and the expression of its downstream gene VEGF by downregulating ARNT, an essential cofactor for HIF-1 [47]. This suggests that, within specific pathways, estrogen signaling may suppress rather than promote hypoxic adaptive responses, revealing a more nuanced and bidirectional regulatory network in processes such as angiogenesis. Conversely, androgens may promote PAI-1 expression. Studies have shown that in prostate cancer tissues, the expression level of the androgen receptor (AR) is significantly positively correlated with the expression of HIF-1α, HIF-2α, and their key downstream effector, VEGF [48]. This indicates that AR signaling may enhance or synergize with HIF-α activity to promote tumor angiogenesis and adaptation to the hypoxic microenvironment.

### Impact of common comorbidities

#### Metabolic diseases

Obesity is a potent stimulus for the overproduction of PAI-1. Research has found that in obese individuals, the mRNA expression level of PAI-1 in abdominal subcutaneous adipose tissue (but not in femoral fat) is significantly positively correlated with both plasma PAI-1 concentration and the degree of systemic insulin resistance. Importantly, following dietary intervention leading to an average weight loss of 9%, PAI-1 gene expression in abdominal subcutaneous fat significantly decreased, accompanied by improvements in metabolic parameters [49].

#### Hypertension

Persistent hypertension potently upregulates PAI-1 expression via the renin-angiotensin system, with angiotensin II serving as a key inducer in this process. Notably, individual genetic differences influence the PAI-1 responsiveness to angiotensin II. Studies indicate that hypertensive patients carrying the PAI-1 4G/4G genotype exhibit a more pronounced increase in PAI-1 levels upon angiotensin II stimulation [50]. This suggests that genetic predisposition and systemic factors jointly determine the elevated PAI-1 state and potential thrombotic risk in hypertensive patients.

## Clinical Applications and Translational Research

### Clinical applications and translational research of HIF-1α

HIF-1α represents a pivotal therapeutic target across multiple disease states. In oncology, HIF-1α overexpression correlates with solid tumor invasion, metastasis, chemo-radioresistance, and poor prognosis [51]. Pharmacological inhibitors or genetic silencing of HIF-1α suppress tumor angiogenesis and glycolytic metabolism [52]. Furthermore, HIF-1α mediates immune evasion through PD-L1 upregulation, suggesting potential synergy with immune checkpoint inhibitors [53]. For ischemic conditions, HIF-1α activators (e.g., roxadustat) ameliorate perfusion deficits in myocardial infarction and limb ischemia models by inducing VEGF and EPO expression [54]. Hypoxic preconditioning of mesenchymal stem cells enhances their paracrine-mediated angiogenic potential in ischemic tissues [55]. In chronic kidney disease, pharmacological HIF-1α stabilization improves renal anemia by stimulating erythropoiesis [56]. Additionally, HIF-1α modulates inflammatory pathogenesis through regulation of macrophage polarization states [57].

### Clinical applications and translational research of PAI-1

PAI-1 represents a critical therapeutic target across thrombotic, fibrotic, oncologic, and metabolic pathologies. Elevated PAI-1 levels establish a hypercoagulable state, correlating with VTE, atherogenesis, and metabolic syndrome while serving as a prognostic biomarker for adverse outcomes [58]. In fibrotic disorders, PAI-1 drives pulmonary, hepatic, and renal fibrogenesis through matrix metalloproteinase (MMP) inhibition [59], with inhibitors like TM5275 demonstrating significant antifibrotic efficacy in preclinical models [60]. Within tumor microenvironments, PAI-1 facilitates obesity-associated oncogenesis by enhancing integrin-mediated collagen I (COL1) engagement and pathological ECM remodeling [61]. In metabolic diseases, pathologically elevated PAI-1 mediates adipose tissue fibrosis and chronic inflammation in obesity and type 2 diabetes mellitus, positioning it as a promising intervention target for metabolic syndrome [62]. Collectively, these findings establish PAI-1 as a multifunctional mediator in thrombosis, fibrosis, cancer progression, and metabolic dysregulation [63].

### Regulatory strategies based on existing clinical drugs

#### Drugs targeting metabolic diseases

Metformin, a first-line medication for type 2 diabetes, exerts pleiotropic protective effects partly through modulating the HIF-1α signaling pathway. Studies have shown that in models of sepsis-associated lung injury, metformin effectively inhibits the accumulation of HIF-1α protein and its mediated aerobic glycolysis by activating the AMP-activated protein kinase (AMPK) pathway [64]. This mechanism suggests the potential of metformin, via AMPK regulation of the HIF-1α axis, to exert protective effects in hypoxic or inflammatory conditions.

#### Lipid-lowering and cardioprotective drugs

Beyond cholesterol reduction, statins exhibit pleiotropic properties. Clinical meta-analyses have confirmed that statin treatment significantly reduces plasma levels of PAI-1 [65], a mechanism potentially linked to the inhibition of Rho protein family signaling [66]. Furthermore, statins improve vascular function by suppressing the RhoA/ROCK pathway [67]. Theoretically, this pathway is also a key regulator of HIF-1α stability under hypoxic conditions, hinting at a potential additional mechanism underlying statin-induced plaque stabilization and improved prognosis.

#### Drugs targeting renal anemia

HIF prolyl hydroxylase inhibitors (e.g., roxadustat, vadadustat) stabilize HIF-α, mimicking the hypoxic response, and effectively stimulate endogenous EPO production. High-level evidence-based medical studies demonstrate that roxadustat shows significant efficacy in correcting hemoglobin levels in patients with chronic kidney disease-associated anemia, even surpassing traditional erythropoiesis-stimulating agents [68]. However, a critical question requiring long-term clinical monitoring is whether the systemic elevation of HIF-α levels by these drugs may potentially influence PAI-1 expression and thrombotic risk.

### Emerging therapeutic agents targeting HIF-1α and PAI-1

#### Agents targeting HIF-1α

Direct pharmacological inhibition of HIF-1α has been pursued as a strategy to disrupt the adaptive responses of tumors and other diseases to hypoxia.

PX-478: A potent and selective small-molecule inhibitor of HIF-1α [69]. It functions by reducing HIF-1α protein levels and inhibiting its transcriptional activity, independent of tumor suppressor genes like VHL and p53 [70]. Preclinical studies have demonstrated its efficacy in sensitizing various solid tumors to other therapies. Notably, recent research highlights that PX-478 can overcome HIF-1α-driven resistance to a novel form of cell death (cuproptosis) in solid tumors [71]. Beyond oncology, PX-478 has shown promise in preserving pancreatic β-cell function in mouse models of diabetes, suggesting potential therapeutic repurposing [72]. While it has entered phase I clinical trials for advanced cancers, further development has faced challenges.

#### Agents targeting PAI-1

Inhibiting PAI-1 activity aims to restore fibrinolytic balance and counteract its pathological roles in fibrosis and tumor progression.

TM5275: An orally bioavailable small-molecule PAI-1 inhibitor [73]. It has been shown to enhance fibrinolysis on vascular endothelial cells by preventing PAI-1 from complexing with tPA [74].

TM5614: Another oral PAI-1 inhibitor under investigation. Preclinical evidence suggests that it can reverse resistance to PD-1 immunotherapy in non-small cell lung cancer models, and it is currently being evaluated in a phase II clinical trial (NCT05015960) [75].

MDI-2268: A potent small-molecule PAI-1 inhibitor effective both *in vitro* and *in vivo* [76]. Studies demonstrate that in disease models, MDI-2268, as a PAI-1 inhibitor, can promote a shift in the immune microenvironment towards a pro-inflammatory state, thereby proving its bioactivity and therapeutic potential in modulating inflammatory responses [77].

### Challenges and directions in translational research

HIF-1α demonstrates context-dependent duality: in ischemic pathologies, its activation promotes angiogenesis and cytoprotection, whereas in tumor microenvironments it facilitates oncogenic progression. This necessitates development of tissue-targeted delivery systems to spatially regulate its beneficial versus detrimental effects. Similarly, PAI-1 exhibits functional dichotomy, promoting hypercoagulable states in thrombosis (VTE, atherosclerosis, metabolic syndrome) while potentially conferring hemostatic protection in bleeding disorders. Such dualistic pathophysiology mandates precise patient stratification for therapeutic interventions. Current translational research focuses on integrating cutting-edge multi-omics approaches, including single-cell sequencing, spatial transcriptomics, and AI-driven predictive modeling, to deconvolute the HIF-1α/PAI-1 regulatory networks and optimize personalized therapeutic strategies. However, clinical translation of HIF-1α/PAI-1-targeted agents faces significant challenges: limited bioavailability, off-target effects, and unresolved long-term safety concerns remain critical barriers [78]. Systematic resolution of these limitations is essential for clinical implementation of effective targeted therapies.

## Challenges and Future Directions

Current mechanistic research on HIF-1α and PAI-1 faces notable constraints. Tissue-specific heterogeneity and dynamic changes across disease stages limit the generalizability of findings, as both factors exhibit context-dependent functional variation. Moreover, interspecies discrepancies between animal models and human pathophysiology restrict translational relevance, compounding research complexity. Regarding therapeutic translation, HIF-1α/PAI-1-targeted agents encounter significant barriers including off-target effects and safety concerns, necessitating further pharmacological optimization to mitigate adverse events. Concurrently, biomarker validation requires multicenter cohort studies to establish robust clinical applicability. Emerging technologies present transformative opportunities in this field. Single-cell resolution analyses can delineate cell subtype-specific regulatory networks, elucidating spatiotemporal mechanisms of HIF-1α and PAI-1. Artificial intelligence-assisted drug design coupled with computational patient stratification enhances therapeutic precision and development efficiency. Strategic integration of these approaches holds promise to: 1) propel fundamental understanding of HIF-1α/PAI-1 biology, 2) address existing translational challenges, and 3) expedite clinical implementation of targeted interventions.

## Data Availability

The authors declare that data supporting the findings of this study are available within the article.
